# NMDA Receptor Antagonists Increase the Release of GLP-1 From Gut Endocrine Cells

**DOI:** 10.3389/fphar.2022.861311

**Published:** 2022-04-27

**Authors:** Malgorzata Cyranka, Thomas Monfeuga, Natascia Vedovato, Chelsea M Larabee, Anandhakumar Chandran, Enrique M Toledo, Heidi de Wet

**Affiliations:** ^1^ Department of Physiology, Anatomy and Genetics, Sherrington Building, University of Oxford, Oxford, United Kingdom; ^2^ Novo Nordisk Research Centre Oxford, Innovation Building, Oxford, United Kingdom

**Keywords:** NMDA receptor (NMDAR), Glucagon-like peptide 1 (GLP-1), GLUTag cells, MK-801 (dizocilpine), dextromethorphan, (DMO), NMDA receptor antagonist

## Abstract

Type 2 diabetes mellitus (T2DM) remains one of the most pressing health issues facing modern society. Several antidiabetic drugs are currently in clinical use to treat hyperglycaemia, but there is a need for new treatments that effectively restore pancreatic islet function in patients. Recent studies reported that both murine and human pancreatic islets exhibit enhanced insulin release and β-cell viability in response to N-methyl-D-aspartate (NMDA) receptor antagonists. Furthermore, oral administration of dextromethorphan, an over-the-counter NMDA receptor antagonist, to diabetic patients in a small clinical trial showed improved glucose tolerance and increased insulin release. However, the effects of NMDA receptor antagonists on the secretion of the incretin hormone GLP-1 was not tested, and nothing is known regarding how NMDA receptor antagonists may alter the secretion of gut hormones. This study demonstrates for the first time that, similar to β-cells, the NMDA receptor antagonist MK-801 increases the release of GLP-1 from a murine L-cell enteroendocrine model cell line, GLUTag cells. Furthermore, we report the 3′ mRNA expression profiling of GLUTag cells, with a specific focus on glutamate-activated receptors. We conclude that if NMDA receptor antagonists are to be pursued as an alternative, orally administered treatment for T2DM, it is essential that the effects of these drugs on the release of gut hormones, and specifically the incretin hormones, are fully investigated.

## Introduction

Recent data suggests that 1 in 10 adults in the United Kingdom will suffer from obesity-associated type 2 diabetes mellitus (T2DM) by 2030, which will reflect a 50% increase in cases when compared to numbers in 2006/2007 (1-in-10 adults living with diabetes by 2030, 2021). The progression of T2DM is characterized by gradually developing peripheral insulin resistance coupled to increased β-cell dysfunction, which ultimately culminates in β-cell death and uncontrolled hyperglycaemia. Multiple T2DM drug classes exist, including some of the more recent drugs presenting cardiovascular benefits such as GLP-1 receptor agonists and gliflozins (SGLT2 inhibitors) (Kahn and Buse, 2015). However, more research needs to be done to develop new treatments that safely and effectively restore β-cell function (Clapham, 2020).

A recent study reported enhanced insulin release from both murine and human pancreatic islets in response to N-methyl-D-aspartate (NMDA) receptor antagonists. Notably, enhanced β-cell viability was demonstrated in both murine and human islets following treatment with NMDA receptor antagonists. Furthermore, the oral administration of dextromethorphan, a NMDA receptor antagonist already in clinical use as a cough suppressant, to type 2 diabetic patients in a small clinical trial resulted in improved glucose tolerance and increased insulin release (Marquard et al., 2016). Another clinical trial showed that the dipeptidyl peptidase-4 (DPP-4) inhibitor sitagliptin improved glucose and insulin levels when low levels of dextromethorphan was co-administered (Marquard et al., 2016). This suggests that NMDA receptor antagonists could be used as adjunct treatment, with a potential benefit on pancreatic cellular functions.

NMDA receptors are glutamate-activated cation channels with high calcium permeability and are heterotetramers comprised of several subunits and subunit splice variants (Paoletti et al., 2013). The role of glutamatergic signalling in neurons is well characterised and, in the brain, glutamate propagates action potentials through two populations of receptors–G-protein-coupled receptors, called metabotropic glutamate receptors (mGluRs), and ligand-gated ion channels, called ionotropic glutamate receptors (iGluRs). NMDA receptors are glutamate- and glycine-activated cation channels that are blocked by Mg^2+^ ions at rest. Mg^2+^ ions are displaced once the membrane is sufficiently depolarised, and NMDA receptors therefore act as so-called coincidence detectors that only propagate action potentials when both ligands and sufficient depolarisation are present simultaneously. The mechanism underlying NMDA receptor regulation of insulin release from pancreatic β-cells is less well understood, but data suggest that NMDA receptor function in the pancreatic islet is inhibitory (Otter and Lammert, 2016). Opposite to the brain, NMDA receptors would therefore have to promote repolarisation of pancreatic β-cells that will close voltage-gated Ca^2+^ channels and switch off insulin release. However, it would be prudent to note that the role of glutamate-activated ionotropic receptors in the pancreatic islet is complex. The cells of the pancreatic islet (α-, β- and δ-cells) are well known to express different iGluR subtypes and in addition to NMDA receptors, both kainite and α-amino-3-hydroxy-5-methyl-4-isoxazole propionate (AMPA) receptors have been reported to be involved in the regulation of increased intracellular [Ca^2+^] levels, which precede insulin exocytosis (Inagaki et al., 1995; Weaver et al., 1996)

Little is known about the role of glutamate receptors in the exocytosis of hormones from the enteroendocrine cells of the gut, and the role of NMDA receptors in the release of the incretin hormone GLP-1 has not been studied to date. Here we report the transcriptional profiling of a murine model gut endocrine cell line, GLUTag cells, with a specific focus on glutamate receptor expression, and compare the transcriptome of GLUTag cells with murine and human L-cells. We establish that GLUTag cells are a good model to investigate NMDA receptor activity and, importantly, we demonstrate a role for NMDA receptor signalling in the stimulus-secretion coupling of GLP-1 from gut endocrine cells. Our work shows that, similar to pancreatic β-cells, antagonism of NMDA receptors results in increased GLP-1 release from an enteroendocrine L-cell line.

## Methods

### Cell Culture of GLUTag Cells

GLUTag cells were a kind gift from Prof Daniel Drucker (Toronto). Standardized cell culture protocols for GLUTag cells used in this study are described in detail in Cyranka *et al* (Cyranka et al., 2019).

### Glucagon-Like Peptide-1-1 Secretion Assays

Active GLP-1 secretion from GLUTag cells was measured by fluorescence resonance energy transfer (FRET)-based ELISA (62GLPPEG; Cisbio) as described in Cyranka et al. (2019). To facilitate a high open probability for NMDA receptors, all secretion experiments were done in Mg^2+^-free Krebs buffer (basal buffer) and was prepared as follows: 120 mM NaCl, 5 mM KCl, 2 mM CaCl_2_, 24 mM NaHCO_3_, 15 mM HEPES, 10 µM Glycine, 0.1% bovine serum albumin (BSA, fatty acid free, A6003, Sigma), pH 7.4 at 37°C, similar to the buffer previously used by Marquard et al. (2016). The following additives were used when indicated in the experiments: 10 µM dizocilpine (MK-801), 100 µM glutamic acid, 300 µM glycine, 5.6 mM glucose, and 10 mM glucose. All experiments were done on ice to minimize GLP-1 degradation. Cell viability in the presence of different buffer conditions and additives were confirmed by Neutral Red cell viability assay (Abcam, ab234039), performed according to the manufacturer’s instructions.

### siRNA Knock-Down of NMDA receptor (*Grin1)* in GLUTag Cells

An overnight culture of GLUTag cells (0.6 × 10^5^ cells/well) in 24-well plates coated with 0.4% Matrigel™(Corning) was transfected with siRNA targeting *Grin1* (probe ID: 62062; AM16708, Ambion). The siRNA complex was prepared according to manufacturer’s protocol with Lipofectamine RNAiMAX (Life Technologies). The amount of siRNA used per well was 600 nM (15 pmol), and 1.5uL of Lipofectamine. The negative control was non-targeting siRNA sequence Silencer Negative Control No. 1 (AM4611, Life Technologies). After transfection, cells were kept at 37°C, 5% CO_2_ for up to 72 h s.

### Total Protein Measurements, SDS-PAGE and Western Blot

Total protein in whole cell lysates of GLUTag cells was quantified by BCA (Bicinchoninic Acid) Protein Assay (Pierce™ BCA Protein Assay Kit - Thermo Fisher Scientific) according to manufacturer’s protocols, and final protein concentrations were calculated using a bovine serum albumin (BSA) standard curve supplied with the kit. Lysate samples were stored at −80°C until Western blot analysis.

SDS-PAGE gel electrophoresis and Western blot analysis were performed according to standard laboratory protocols. The anti-NMDAR1/Grin1 primary antibody was AB9864, rabbit mAB from Merck-Millipore, visualized by A16035 donkey anti-rabbit IgG:HRP from Life Technologies. Western blots were quantified using Fiji (NIH) and normalised to the loading control for each lane.

### Statistics

For cell culture studies, data are presented as mean ± standard deviation. Data was analysed by repeated measures one-way ANOVA or multiple t-tests (Prism) using a Holm-Šídák’s post-hoc analysis multiple comparison correction. A *p* value ≤ 0.05 for a 95% confidence interval was regarded as statistically significant. Statistics were performed using GraphPad Prism6 (Graphpad Software, CA, United States). GLP-1 secretion assays are shown as three experimental data points or more, with each experimental data point representing the mean of three technical repeats, unless otherwise indicated in the legends to the figures. Experiments were performed on cells from different frozen stocks and different cell passages to control for interbatch variation. A total of 6–24 data points from the technical repeats were pooled and analysed for normal distribution using Prism, and normality of the data was confirmed.

### Transcriptome (mRNA) Library Preparation and Sequencing

GLUTag cells were cultured in low glucose (1g/L) DMEM (Cat.no. 11880028, Life Technologies) supplemented with GlutaMAX (Cat.no. 35050061, Life Technologies) and 10% in-house heat inactivated FBS (Cat.no. F7524, Sigma). Cells were seeded at 1 × 10^6^cells/well in 6-well dishes coated with 0.4% Matrigel (BD, Cat.no. 354234) and grown for 48 h s until the cell harvest. mRNA was isolated using a Dynabeads mRNA DIRECT Kit (Cat.no. 61012, Invitrogen™). Extracted mRNA was used to prepare Illumina compatible sequencing libraries with QuantSeq 3′mRNA-Seq Library Prep Kit-FWD (Cat.no. 15, Lexogen) based on the manufacturer’s instructions. The final sequencing libraries were pooled and sequenced in Illumina NextSeq 500 system.

### Additional RNA Sequencing Datasets

Publicly available RNA-sequencing datasets of human and murine enteroendocrine cells (Roberts et al., 2019) as well as primary ileal organoid cultures (Goldspink et al., 2020) were retrieved from NCBI’s Gene Expression Omnibus (GEO) (Edgar et al., 2002) under the respective accession numbers GSE114853, GSE114913 and GSE148224. In the dataset GSE148224, cells cultured in both IF and IF* media were included in the analysis due to the similarity of their transcriptome (Goldspink et al., 2020). The FACS-sorted cells from each study were renamed and arranged into groups for the purpose of this paper, as detailed in Table 1.

**TABLE 1:** Dataset and sample description.

Dataset	Species	Original sample name	Sample size	Tissue	New sample name	Group
GSE114853	Human Roberts et al. (2019)	GLP-1^-^/CHGA^−^/SCG2^-^	11	Jejunum	non EEC	non-EEC cells
			2	Ileum		
		GLP-1^-^/CHGA^+^/SCG2^+^	11	Jejunum	EEC GCG NEG	non-L-cell EECs
			2	Ileum		
		GLP-1^+^/CHGA^+^/SCG2^+^	11	Jejunum	EEC GCG POS	L-cells
			2	Ileum		
GSE114913	Mouse Roberts et al. (2019)	GLU-Venus negative	2	Duodenum	GLUVenus NEG	Mix of EEC and non-EEC, but no L-cells
		GLU-Venus positive	3	Duodenum	GLUVenus POS	L-cells
		NeuroD1-cre-eYFP negative	3	Duodenum	NeuroD1 NEG	non-EEC-cells
		NeuroD1-cre-eYFP positive	3	Duodenum	NeuroD1 POS	Total EEC population
GSE148224	Human Goldspink et al. (2020)	Venus negative	8	Ileal organoid	GLUVenus NEG	Mix of EEC and non-EEC, but no L-cells
		Venus positive	8	Ileal organoid	GLUVenus POS	L-cells

CHGA, chromogranin A; ECC, enteroendocrine cell; GLP-1, glucagon-like peptide 1; SCG2, secretogranin II (chromogranin C); GCG, (prepro)glucagon.

### RNA Sequencing Analysis

Quality control and adaptor trimming of the sequencing data were performed with FastQC (v. 0.11.9) and Trim Galore! (v. 0.6.4_dev). Salmon (v. 1.5.1) (Patro et al., 2017) was used to align reads to decoy-aware indexes built using transcript sequences and primary assembly genomes from Gencode (human data: GRCh38, release 38; mouse data: GRCm38, release M23). For the QuantSeq 3′ mRNA sequencing data, the --noLengthCorrection argument was used. Alignment statistics were collected with MultiQC (v. 1.11) (Ewels et al., 2016). The average mapping rate of the newly sequenced GLUTag dataset was 66% (standard deviation (s.d.) 4%), leading to an average number of mapped reads of 27.3 million (M) (s.d. 5.8 M). For the publicly available datasets GSE114853, GSE114913 and GSE148224, these statistics were, respectively, 23% (s.d. 8%), 2.5 M (s.d. 1.4 M); 62% (s.d. 3%), 6.2 M (s.d. 4.3 M); 29% (s.d. 7%), 8.5 M (s.d. 2.8 M). Transcript quantification import, transcript-per-million (TPM) transformation (whenever relevant) and gene-level summarizations were performed by the R package Tximeta (v. 1.10) (Love et al., 2020), using the countsFromAbundance = “no” argument.

In order to compare the human and mouse transcriptomes, datasets were merged using one-to-one orthologues pairs using Ensembl data (release 102) (Howe et al., 2021) from BioMart (v. 2.46.3) (Smedley et al., 2009). To minimize the batch effect of sequencing technologies and independent studies, the ComBat_seq() function of the R package sva (v. 3.38.0) (Leek et al., 2012; Zhang et al., 2020) was used, defining the datasets as batches. Subsequently, the corrected count data was normalized by library size and variance-stabilizing transformed using the vst() function of the R package DESeq2 (v. 1.30.1) (Love et al., 2014). Normalized counts were averaged within each group to be compared and the Pearson correlations’ R-squared values calculated between each group (All R values were positive before squaring). The results were then plotted as a heatmap using the default parameters (complete linkage hierarchical clustering using the Euclidean distances) of the pheatmap function of the R package ComplexHeatmap (v. 2.6.2) (Gu et al., 2016). BioMart annotations and the IUPHAR/BPS Guide to PHARMACOLOGY complete “target and family” list (v. 2021.3) were used to restrict the analyses to specific gene types and families (Harding et al., 2022). For within-dataset comparisons (gene expression plots), non-batch corrected transcript-per-million (TPM) values were used (corresponding to count-per-million (CPM) for the QuantSeq 3′ mRNA sequencing data).

Data processing was performed in R (v. 4.0.5) using the tidyverse packages (v. 1.3.1). Plots were generated using combinations of the packages ComplexHeatmap (v. 1.42.3), ggplot2 (v. 3.3.5), ggpubr (v. 0.4.0), lemon (v. 0.4.5), RColorBrewer (v. 1.1–2), cowplot (v. 1.1.1) and ggplotify (v. 0.1.0).

### Data and Code Availability

The GLUTag-related sequencing data generated and discussed in this publication has been deposited and is available under the GEO accession number GSE193866. The R code used to generate the results is available in the github repository https://github.com/novonordisk-research/GLUTag_analysis.

## Results

### Antagonism of NMDA Receptors Leads to Increased Release of GLP-1 From GLUTag Cells

**FIGURE 1:**
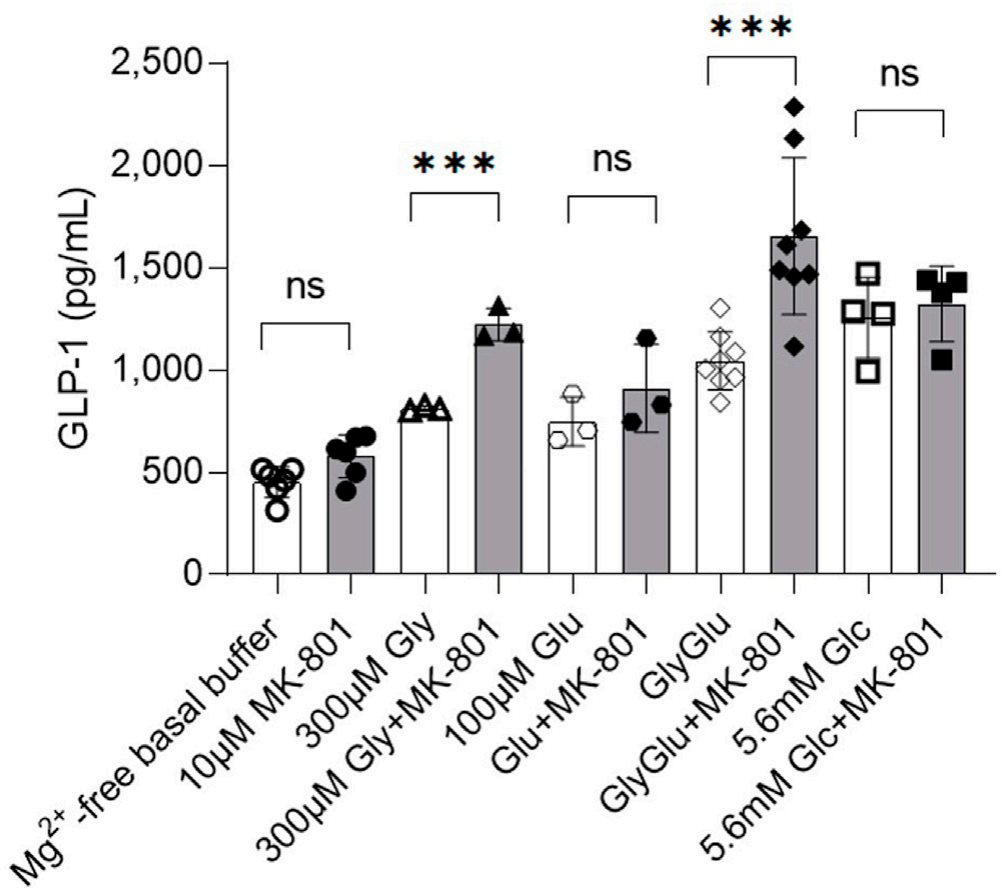
Release of GLP-1 from cultured GLUTag cells incubated for 2 h with (filled/grey) or without (open/white) NMDAR antagonist MK-801 and glycine (triangles), glutamate (hexagons), glycine and glutamate (diamonds), or glucose (squares). Data shown as mean ± SD, n = 3–8; ***p < 0.001, ns = not significant by grouped unpaired *t*-tests with Holm-Šídák’s multiple comparisons correction. Gly, glycine; Glu, glutamate; Glc, glucose.

The addition of the non-competitive NMDA receptor antagonist MK-801 (10 μM) for 2 h to GLUTag cells cultured in buffer containing glycine with glutamate showed a 37% increase in GLP-1 release relative to control samples (Figure 1A, open diamonds, 1,045 ± 142 pg GLP-1/ml vs. filled diamonds, 1,655 ± 382 pg GLP-1/ml), while buffer containing glycine alone showed a 34% increase in GLP-1 release (Figure Fig1A, open triangles, 812 ± 12 pg GLP-1/ml vs. filled triangles, 1,223 ± 80 pg GLP-1/ml) when compared to controls. The stimulatory effect of MK-801 was not significant in the presence of basal buffer in the absence of Mg^2+^ ions (Figure 1A, filled circles), glutamate (Figure 1A, hexagons) or in the presence of 5.6 mM glucose (Figure 1A, squares). The data are consistent with the effect of NMDA receptor antagonism being most prominent in Mg^2+^-free buffer in the presence of both the NMDA receptor agonists, glutamate and glycine, as these buffer conditions would ensure the highest channel open probability for NMDA receptor. GLUTag cell viability were not affected by the buffer conditions or additives used in this study (Supplementary Figure S1) and GLP-1 secretion under basal buffer conditions was not affected by the presence (460 ± 102 pg GLP-1, n = 6) or absence (505 ± 17 pg GLP-1/ml, n = 6) of Mg^2+^. Unfortunately, these studies could not be extrapolated to *ex vivo* primary gut crypt cultures as these cultures were not compatible with Mg^2+^-free buffer and established crypts detached rapidly in the absence of Mg^2+^ ions.

### siRNA Knockdown of *Grin1* in GLUTag Cells Increase GLP-1 Release

**FIGURE 2:**
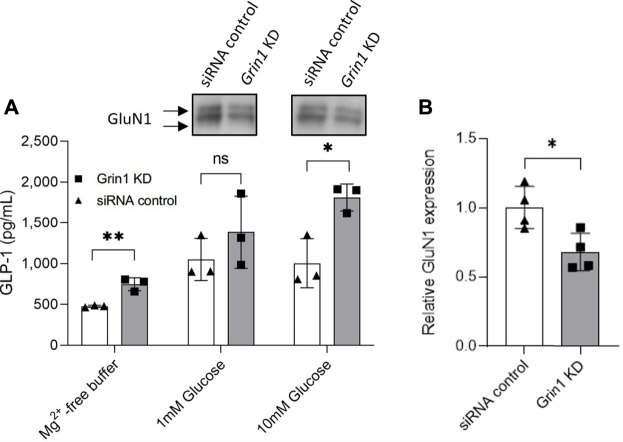
**(A)** Release of GLP-1 from cultured GLUTag cells incubated for 72 h with *Grin1*-specific siRNA (squares/grey) or control siRNA (triangles/white) with 0, 1, or 10 mM glucose. Insets show western blots from cell lysates using a general NMDAR1/GluN1 antibody. **(B)** Quantitation of western blot analysis of GluN1 expression in GLUTag cells incubated for 72 h with *Grin1*-specific siRNA (squares/grey) or control siRNA (triangles/white). Data shown as mean ± SD, n = 4; *p = 0.05, **p < 0.02, ns = not significant by **(A)** grouped unpaired *t*-tests with Holm-Šídák’s multiple comparisons correction, n = 3 or **(B)** paired *t*-test, n = 4.

In order to control for any possible off-target effects of MK-801—which is known to also target acetylcholine receptors (nAChR), and to unequivocally confirm a direct role of NMDA receptor activity in the regulation of GLP-1 release from GLUTag cells, the essential NMDA receptor subunit, GluN1 (also called NMDAR1 or zeta-1), was knocked down by siRNA targeting of the gene *Grin1,* (Figure 2; Supplementary Figure S2). Western blot analysis showed a significant decrease in GluN1 protein expression levels in siRNA-treated GLUTag cells (Figure 2A, WB inserts and Figure 2B, 32% ± 10.5%). This decrease in protein expression levels coincided with up to 31% increased GLP-1 release from siRNA-treated GLUTag cells (Figure 2, squares, 746 ± 79 pg GLP-1/ml, 1,385 ± 444 pg GLP-1/ml, and 1812 ± 166 pg GLP-1/ml for Mg^2+^-free buffer, 1 mM glucose, and 10 mM glucose, respectively) when compared to control siRNA probe (Figure 2, triangles, 476 ± 13 pg GLP-1/ml, 1,049 ± 257 pg GLP-1/ml, and 1,002 ± 300 pg GLP-1/ml for Mg^2+^-free buffer, 1 mM glucose, and 10 mM glucose, respectively). This effect was most prominent in the presence of 10 mM glucose (1812 ± 166 pg GLP-1/ml Grin1 siRNA vs. 1,002 ± 300 pg GLP-1/ml siRNA control).

### Transcriptional Profiling of GLUTag Cells and Comparison to Murine and Human L-Cells

As *ex vivo* gut crypt cultures were not compatible with buffer conditions that favor a high open probability for NMDA receptors, NMDA receptor expression in GLUTag cells and in both murine and human L-cells were investigated to ascertain if GLUTag cells would be a good model system to investigate NMDA receptor activity. To this effect, RNA-sequencing of GLUTag cells was performed and their transcriptome compared to murine and human cells. Publicly available datasets of FACS-sorted *in-vitro* primary ileal organoid cells (Goldspink et al., 2020) as well as FACS-sorted *in vivo* gastrointestinal cells from both mouse and human gut tissue (Roberts et al., 2019) were downloaded and processed. The different sample types from each study are detailed in Table 1, alongside the related abbreviations used in this paper.

**FIGURE 3:**
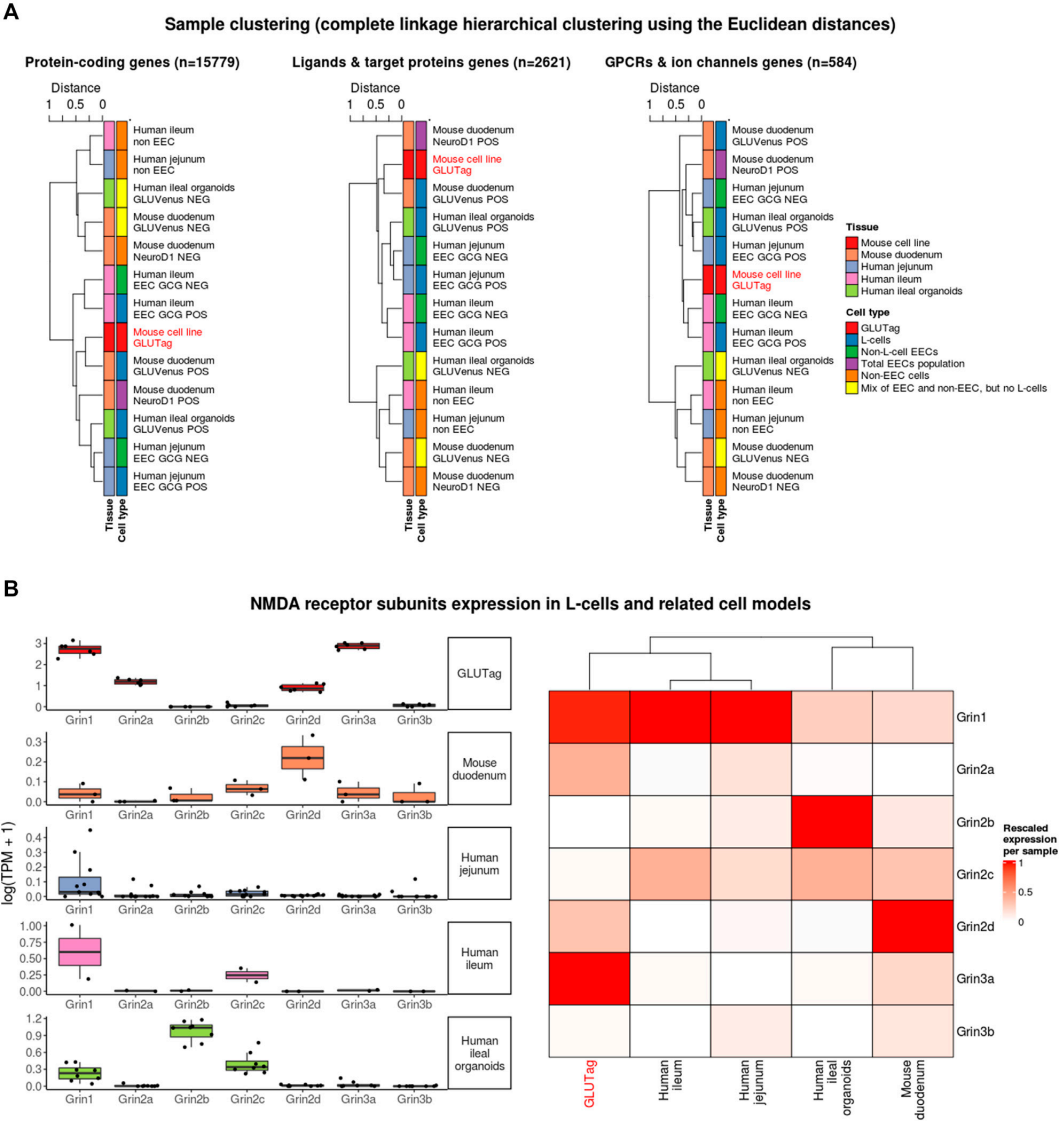
Transcriptome similarity between GLUTag cells and other gastrointestinal samples. **(A)** Hierarchical clustering based on Pearson correlation (*R* ^2^ values) for different subsets of the transcriptomes **(B)** NMDA receptor expression for cultured GLUTag cells and L-cells FACS-sorted from mouse tissue (duodenum), human tissue (jejunum and ileum) and human ileal organoids. Left side: absolute values represented as log-transformed transcripts per million (TPM); right side: relative expression after rescaling per sample (1 = most expressed NMDA receptor subunit in the sample) and clustering.

Using Pearson correlations and clustering of the *R* ^2^ values, the datasets were used to determine which enteric cell transcriptomes were the most strongly correlated with the transcriptome of GLUTag cells. Merging of the datasets, using variance-stabilized normalized data, led to a clustering by species and datasets (data not shown). This is partially due to the different sequencing technologies and protocols used for each independent dataset. To correct for the batch effects due to the different methods utilized (using GEO datasets as batch), the software Combat-Seq (Zhang et al., 2020) was used to observe transcriptome correlations between different tissues/cell types rather than between studies/technologies (as in (Gilad and Mizrahi-Man, 2015)). A principal component analysis showed that the main variance in the resulting data was driven by differences in cell types (Supplementary Figure S3). Moreover, genes contributing the most to the first principal component are relevant to the cells’ functions, such as their secretory phenotype (e.g., Gcg, Gip, Pyy). After this correction, all sample groups were clustered using genes from three subsets of the transcriptomes: protein coding genes (n = 15779), genes coding for ligands and target proteins (using the IUPHAR/BPS Guide to PHARMACOLOGY, “an expert-curated resource of pharmacological targets and the substances that act on them” (Harding et al., 2022)) (n = 2,621) or genes coding for G-protein-coupled receptors (GPCRs) and ion channels (n = 584). In all three cases, the transcriptome of GLUTag cells was observed to be most similar to entero-endocrine cells (regardless of species/tissues) compared to samples containing non-EEC cells (Figure 3A), although the GLUTag cells seem to preferentially cluster with human ileal cells rather than the mouse duodenal cells when restricted to GPCRs and ion channel genes (Figure 3A; Supplementary Figure S4).

Next, the expression data of the NMDA receptor subunit genes were specifically analysed. In GLUTag cells, the two main NMDA receptor subunits expressed are Grin3a and Grin1 (Figure 3B). After rescaling the expression of these genes within L-cells and L-cell models (GLUTag and GLUVenus POS organoid cells), GLUTag cells clustered with human L-cells due to the similarly high expression of Grin1 relative to other NMDA receptor subunit genes.

## Discussion

Stimulation-secretion coupling in L-cells, and specifically GLUTag cells, is well characterised (Gribble and Reimann, 2021). Unlike pancreatic β-cells, in which insulin secretion is mainly coupled to circulating glucose levels, enteroendocrine cells evolved to secrete hormones in response to the large variety of different compounds typically found in digested chyme, including glucose, fatty acids, amino acids and bile. Although glucose-stimulated GLP-1 release is coupled to membrane depolarization through SGLT-1 co-transporter function and voltage-gated Ca^2+^ channels, GPCR pathway activation and cAMP-mediated regulation of exocytosis also play major roles in coupling GLP-1 with the arrival of fatty acids, amino acids and bile in the small intestine (Yan et al., 2022).

Little is known about the role of glutamate receptor signaling in gut enteroendocrine cells, but it would appear that, similar to NMDA receptors in pancreatic β-cells, NMDA receptor activity also inhibits GLP-1 release from L-cells. This study demonstrates that both antagonism or siRNA knock-down of the L-cell NMDA receptor results in increased GLP-1 release. Notably, this effect was independent of glucose-dependent membrane depolarization, as increased GLP-1 release in response to siRNA knock-down of the essential NMDA receptor subunit GluN1 was present in both basal Mg^2+^-free buffer conditions and buffer containing glucose. In agreement with a role for NMDA receptors in GLP-1 release, the stimulatory effect was most prominent in the presence of the essential agonists glycine and glutamate. However, increased GLP-1 release in response to MK-801 was not observed in the presence of glutamate alone, while glycine-only buffer did show MK-801 stimulated GLP-1 release. It has been demonstrated that glutamate is co-released with GLP-1 from GLUTag cells, which could explain the increased GLP-1 release in response to MK-801 in glycine-only buffer, as released glutamate will most likely be available to activate NMDA receptors (Uehara et al., 2006). Furthermore, as GLUTag cells are maintained in the presence of 10% FBS, which typically contains mM amounts of both glycine and glutamate, it is possible that some active NMDA receptors may remain when cells are transferred to glycine- and/or glutamate-free buffers (Elhassan et al., 2001).

In summary, similar to pancreatic β-cells, we appear to unmask an inhibitory function for NMDA receptors in L-cells, where loss-of-function of active NMDA receptors results in increased hormone exocytosis. It would therefore appear that NMDA receptor activity acts as a general “hand-brake” to regulate GLP-1 release from L-cells, and by extension, would act to repolarise membrane potentials following depolarisation events triggered by the arrival of nutrients in the gut. However, it is well established that enteroendocrine cells form direct, physical contacts with extrinsic enteric-associated neurons (both the sensory afferents and autonomic efferents) which innervate the gut and directly link the gut to the brain via the vagus nerve (Bohórquez et al., 2015; Kaelberer et al., 2018). The possibility that NMDA receptors could play a role in the regulation of glutamatergic neurotransmission in intact gut tissue should therefore also be considered.

3′-mRNA sequencing reveals that GLUTag cells appear to be a good model to investigate the function of NMDA receptors in human gut endocrine cells. NMDA receptors are heterotetramers composed of two GluN1 (NR1) subunits combined with two NR2A-D or NR3A-B subunits to form a functional channel. There are differences in NDMA receptor subunit composition of GLUTag and human enteroendocrine cells and the most abundant NMDA receptor subunit genes in GLUTag cells are *Grin3a* and *Grin1*, while *Grin2b* is most abundant in human ileal organoids. Both GLUTag cells and human enteroendocrine cells express high levels of *Grin1* relative to other NMDA receptor subunit genes, which would suggest that NMDA receptor function is conserved in both these cells. The overall similarity of the GLUTag transcriptome compared to human and murine enteric cells was also investigated. The main limitation of the analysis performed is a result of the confounding factors of the varied technologies and methodologies used to generate data between studies. For this reason, it is not possible to differentiate which differences in the transcriptomes are due to biological or non-biological effects without a batch correction method. To that effect, batch correction was used in this study to minimize non-biological variance and to compare cell types more efficiently, with the drawback of potentially losing some species-specific (biological) differences. This correction allowed us to ascertain which cell type the GLUTag cells were the most similar to within each study. Here, the analysis across various independent datasets shows that the transcriptome of GLUTag cells is closer to other entero-endocrine cells (including L-cells) than the other enteric cells tested, supporting further the use of GLUTag cells as a relevant model to investigate NMDA receptor function.

To conclude, although the use of model cell lines is now being superseded by more sophisticated models such as *ex vivo* cell cultures and organoids, model cell lines remain a low cost and fast alternative to screen the impact of drugs on a wide range of endocrine cells under buffer conditions that may not be well tolerated by *ex vivo* models and organoids. Transcriptional profiling enables confirmation of target expression in the model cell line of interest and also gives an indication to the similarity between the model cell line and organoids from similar tissue origin. This study demonstrates that if existing NMDA receptor antagonists are to be repurposed as an orally administered treatment for T2DM, it is essential that the effects of these drugs on the release of incretin hormones from the gut are fully investigated.

## Data Availability

The GLUTag-related sequencing data generated and discussed in this publication has been deposited and is available under the GEO accession number GSE193866. The R code used to generate the results is available in the github repository https://github.com/novonordisk-research/GLUTag_analysis.
