# Supplementary material for: NMDA Receptor Antagonists Increase the Release of GLP-1 From Gut Endocrine Cells
Source: Front Pharmacol. 2022 Apr 27;13:861311. doi: 10.3389/fphar.2022.861311 (PMC9091448; doi:10.3389/fphar.2022.861311)
Supplement: Supplementary file 1 [file Presentation1.pdf]

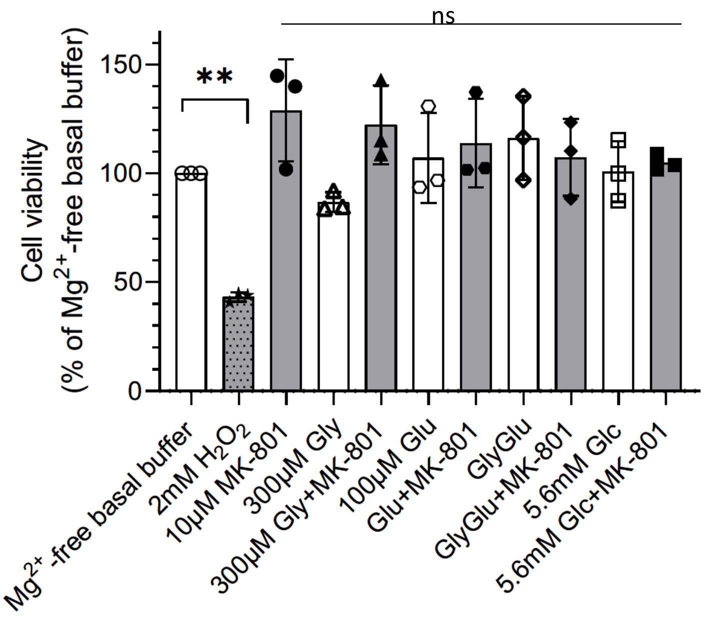

Supplementary Figure 1. Cell viability measured by Neutral Red assay in cultured GLUTag cells incubated in Mg^{2+}-free basal buffer for 2h with (filled/grey) or without (open/white) NMDAR antagonist MK-801; plus glycine (triangles), glutamate (hexagons), glycine and glutamate (diamonds), or glucose (squares). 2mM H_2O_2 as a control for decreased cell viability (dotted bar, stars). Data shown as mean±SD, n=3; **P<0.01. ns=not significantly different from Mg^{2+}-free basal buffer by one-way ANOVA with Holm-Šídák's multiple comparisons correction. Gly, glycine; Glu, glutamate; Glc, glucose.

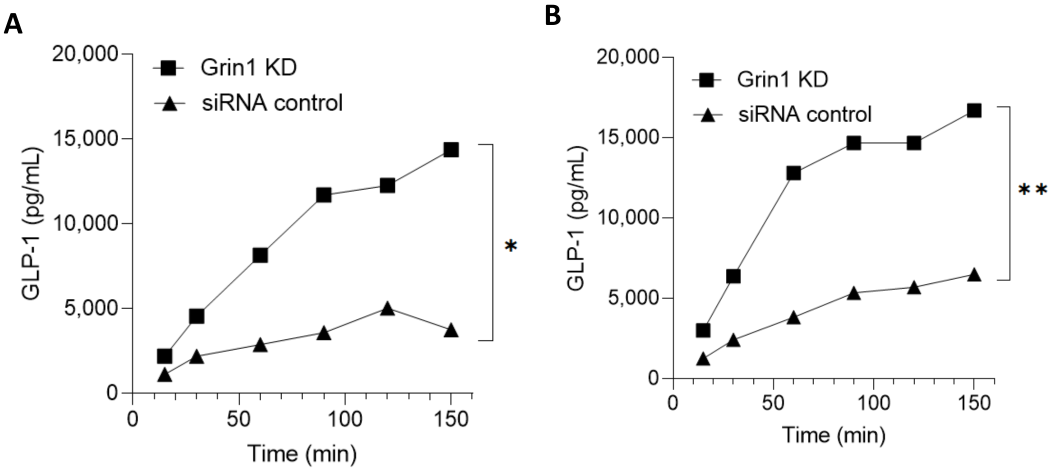

Supplementary Figure 2. Time-dependent release of GLP-1 from cultured GLUTag cells incubated for 72h with *Grin1*-specific siRNA treatment (squares) or control siRNA (triangles) in **(A)** Krebs buffer or **(B)** 1mM glucose, n=1; **P<0.02, ***P<0.001 by repeated measures one-way ANOVA with Holm-Šídák's *post hoc* test.

A

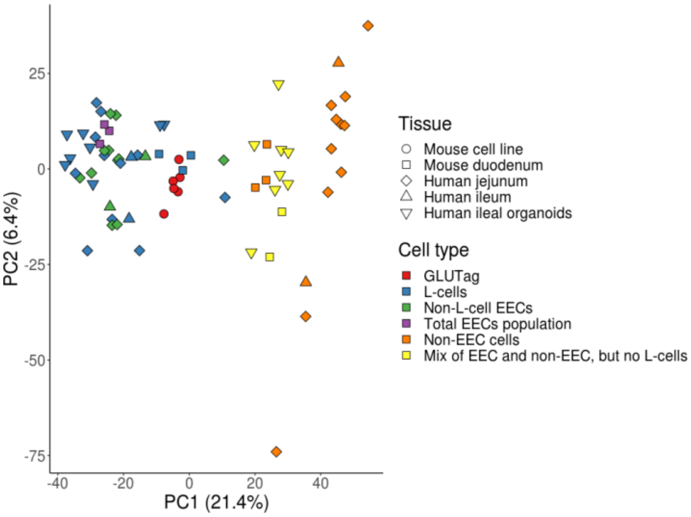

B

#	Top genes: PC1		Top genes: PC2	
1	Gcg	*(0.78%)*	Apob	*(0.34%)*
2	Nts	*(0.6%)*	Obscn	*(0.26%)*
3	Chga	*(0.59%)*	Sis	*(0.23%)*
4	Scg2	*(0.57%)*	Anpep	*(0.23%)*
5	Cck	*(0.55%)*	Muc3a	*(0.22%)*
6	Ucn3	*(0.53%)*	Il7r	*(0.21%)*
7	Pcsk1	*(0.51%)*	Mep1b	*(0.21%)*
8	Cacna1a	*(0.47%)*	Ttn	*(0.19%)*
9	Chgb	*(0.47%)*	Cdh1	*(0.19%)*
10	Gip	*(0.46%)*	Dsp	*(0.18%)*
11	Neurod1	*(0.45%)*	Tspoap1	*(0.17%)*
12	Pcsk1n	*(0.43%)*	Wipf1	*(0.16%)*
13	Vwa5b2	*(0.42%)*	Itgal	*(0.16%)*
14	Scg5	*(0.42%)*	Dock2	*(0.15%)*
15	Rimbp2	*(0.4%)*	Arhgap30	*(0.15%)*
16	Pyy	*(0.4%)*	Spn	*(0.14%)*
17	Scgn	*(0.39%)*	Ccdc88b	*(0.14%)*
18	Cpe	*(0.38%)*	Mme	*(0.14%)*
19	Insm1	*(0.37%)*	Akna	*(0.14%)*
20	Abcc8	*(0.37%)*	Epcam	*(0.14%)*

Supplementary Figure 3. Principal component analysis (PCA) after batch-correction of the integrated datasets. **(A)** PCA plot showing a separation in the first principal component between enteroendocrine and non-enteroendocrine cells. **(B)** Top 20 features (genes) contributing the most to each of the first 2 principal components (PCs). The percentage of the contribution of each gene to the PCs is shown in parentheses.

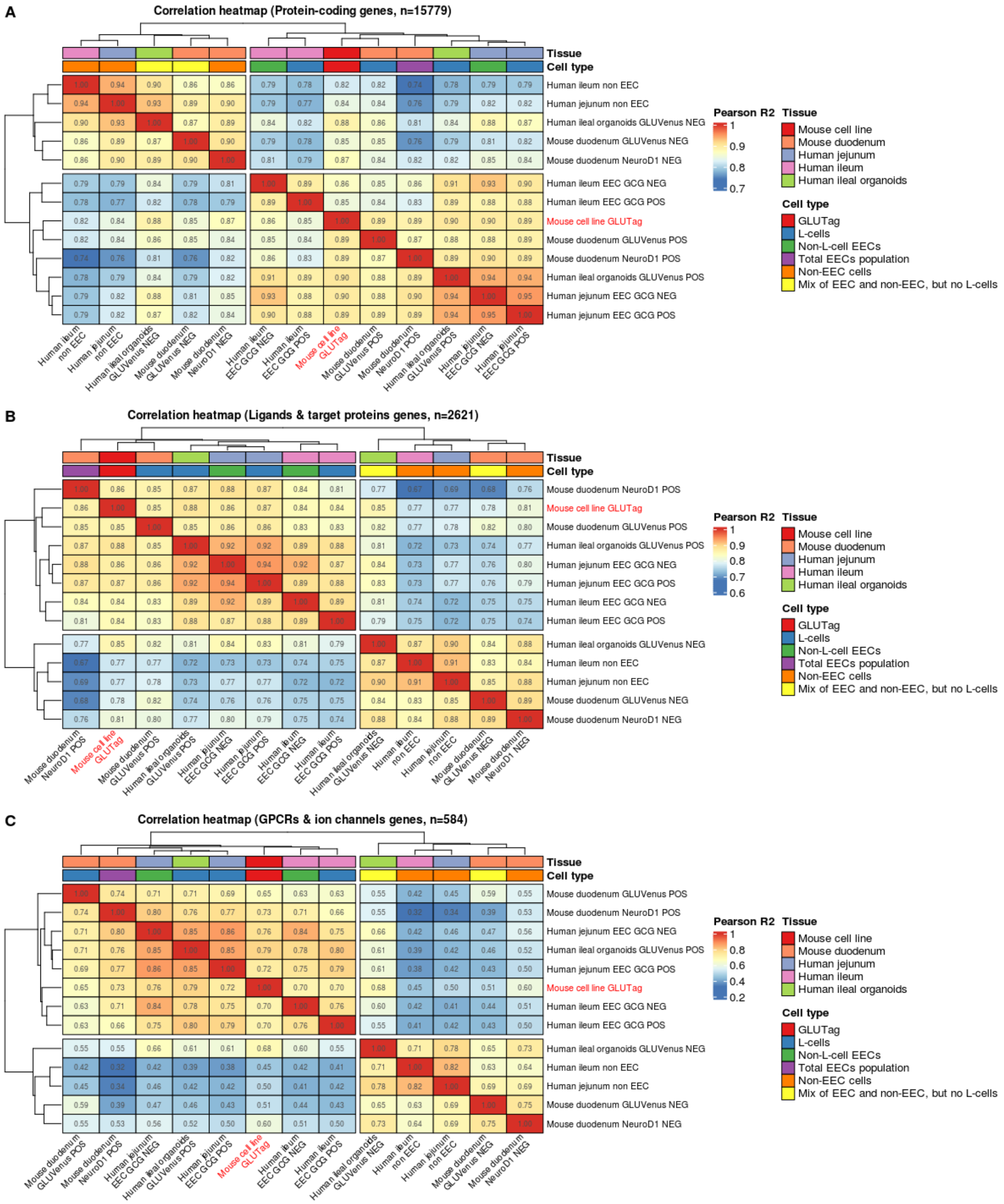

Supplementary Figure 4. Correlation heatmaps between GLUTag cells and **(A)** protein-coding genes, **(B)** ligand and target protein genes (list from the IUPHAR/BPS Guide to PHARMACOLOGY, a curated database of molecular interactions between ligands and their targets) and **(C)** GPCR and ion channel genes. The inter-sample correlations between the normalised expression values of each transcriptome was calculated and used for clustering (complete linkage using Euclidean distances between the Pearson correlation R^2 values).

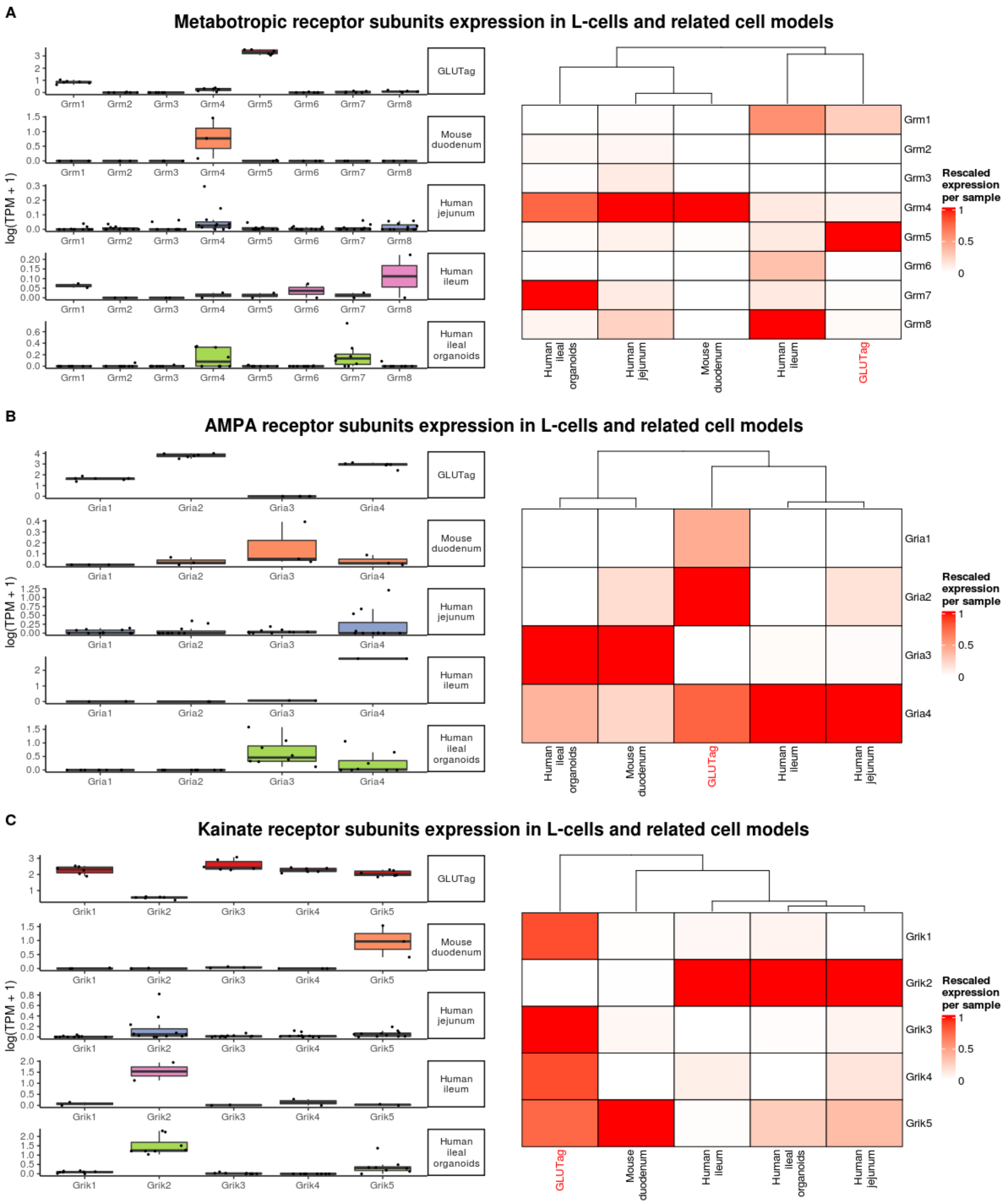

Supplementary Figure 5. Gene expression of selected glutamate receptor genes for GLUTag cells and FACS-sorted L-cells from mouse tissue (duodenum), human tissue (jejunum and ileum) and human ileal organoids. Left side: absolute expression values represented as log-transformed transcripts per million (TPM); right side: relative expression after rescaling per sample (1 = most expressed receptor subunit in the sample) and clustering.
